# ApoE4 exacerbates the senescence of hippocampal neurons and spatial cognitive impairment by downregulating acetyl‐CoA level

**DOI:** 10.1111/acel.13932

**Published:** 2023-08-18

**Authors:** Shuixin Lv, Yusi Zhang, Yingbin Lin, Wenting Fang, Yu Wang, Zihang Li, Anlan Lin, Xiaoman Dai, Qinyong Ye, Jing Zhang, Xiaochun Chen

**Affiliations:** ^1^ Department of Neurology Fujian Medical University Union Hospital, Fujian Key Laboratory of Molecular Neurology and Institute of Neuroscience, Fujian Medical University Fuzhou China; ^2^ School of Basic Medical Sciences Fujian Medical University Fuzhou China; ^3^ Department of Pathology the First Affiliated Hospital of Fujian Medical University Fuzhou China

**Keywords:** acetate, acetyl‐CoA, Alzheimer's disease, ApoE4, synaptic plasticity

## Abstract

Although aging and apolipoprotein E (*APOE*) ε4 allele have been documented as two major risk factors for late‐onset Alzheimer's disease (LOAD), their interaction and potential underlying mechanisms remain unelucidated. Using humanized ApoE4‐ and ApoE3‐ target replacement mice, we found the accumulation of senescent neurons and the activation of mTOR and endosome‐lysosome‐autophagy (ELA) system in the hippocampus of aged ApoE4 mice. Further analyses revealed that ApoE4 aggravated the profile change of hippocampal transcription and metabolism in an age‐dependent manner, accompanying with an disruption of metabolism, which is presented with the downregulating activity of citrate synthase, the level of ATP and, most importantly, the level of acetyl coenzyme A (Ac‐CoA); GTA supplement, an Ac‐CoA substrate, reversed the senescent characteristics, decreased the activation of mTOR and ELA system, and enhanced the synaptic structure and increasing level of pre‐/post‐synaptic plasticity‐related protein, leading to cognitive improvement in aged ApoE4 mice. These data suggest that ApoE4 exacerbates neuronal senescence due to a deficiency of acetyl‐CoA, which can be ameliorated by GTA supplement. The findings provide novel insights into the potential therapeutic value of GTA supplement for the cognitive improvement in aged *APOE*4 carriers.

## Graphical abstract

Elderly ApoE4 mice exhibits a greater number of senescent neurons and lower level of Ac‐CoA compared with those elderly ApoE3 mice, accompanying with elevated levels of p53, mTOR and ELA system, and poorer synaptic plasticity. While supplementary of acetate, both of E3 and E4 show high level of Ac‐CoA. ApoE4 mice could show lower senescent makers as described above, and synaptic plasticity also get improved. Furthermore, both of E3/E4 mice with acetate treatment show better cognitive performance in memory test.

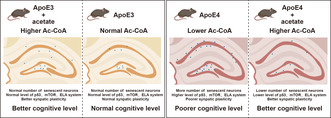

## 1 INTRODUCTION

In aging‐related neurodegenerative diseases (NDs), cumulative evidence suggest that cellular senescence plays a key role in the progression of NDs, including Alzheimer's diseases (AD), Huntington's disease, tau‐related diseases, etc. (Kritsilis et al., 2018; Musi et al., 2018; Voisin et al., 2020) Studies have documented that the clearance of senescent cells prevents neuropathological progression and cognitive decline (Bussian et al., 2018; Chow et al., 2019). In addition to aging, the apolipoprotein E4 (*APOE*4) genotype has been well documented to substantially impact the onset and development of NDs (Hyman et al., 1996; Lane‐Donovan et al., 2016). Studies have pinpointed *APOE*4 as the strongest genetic risk factor for late‐onset AD (LOAD) and the culprit for increasing occurrence of cognitive decline in elderly people (Genin et al., 2011; Liu et al., 2014). However, it remains obscure regarding the involvement of ApoE4 in an aging brain and the underlying mechanisms.

In cellular metabolism, acetyl coenzyme A (Ac‐CoA) is a crucial metabolic intermediate. The abundance of Ac‐CoA reflects the general energetic state of the cells (Ghosh‐Choudhary et al., 2020; Peleg, Feller, Ladurner, & Imhof, 2016). A low level of Ac‐CoA may increase autophagy and directly extend the life span of, via the epigenetic regulation of gene expression, yeast, drosophila (Eisenberg et al., 2014; Peleg, Feller, Forne, et al., 2016) and *Caenorhabditis elegans (*Zhu et al., 2020 *)*; Ac‐CoA administration promote the senescence in yeast and human endothelial cells (Chen et al., 2021). However, it has yet to be illustrated whether the level of Ac‐CoA also affects cellular senescence and autophagy in an aging brain. Meanwhile, Ac‐CoA pools are partially regulated by Ac‐CoA synthetase 2 (ACSS2), which is essential for hippocampal spatial memory in adult mice (Mews et al., 2017). Our recent research also indicates the critical role of ACSS2 in cognitive decline in AD mice (Lin et al., 2023). We hypothesize that ApoE4 may impair the cognitive function due to the Ac‐CoA shortage in the hippocampus of normal aging mice.

In the cellular metabolism, chromatin organization, and transcription, drastic changes occur. For one thing, senescent cells secrete a group of factors, collectively termed as the senescence‐associated secretory phenotype (SASP), which influence the homeostasis of aging tissues and contribute to the development of NDs. For another, the protein synthesis and autophagic degradation are regulated in an opposite manner by mammalian target of rapamycin (mTOR). During oncogene‐induced senescence (OIS), cells augment their secretory phenotypes by coordinating protein synthesis and autophagy in the TOR‐autophagy spatial coupling compartment (TASCC) (Narita et al., 2011). In irradiation‐induced senescence (IR) and OIS, increased autophagy and high levels of intracellular amino acids may boost the mTORC1 activity, which promotes the survival of senescent cells (Bernard et al., 2020; Carroll et al., 2017). Studies of molecular mechanism have suggested that mTOR regulates the mitogen‐activated protein kinases‐activated protein kinase‐2 (MAPKAPK2) translation to control the SASP (Herranz et al., 2015) and that mTOR activation plays a key role in the survival of senescent cells and the maintenance of the senescence phenotype (Perluigi et al., 2015; Tomimatsu & Narita, 2015; Xu et al., 2014). However, the role of mTOR in aging brain tissues awaits further illumination.

In present study, we explored the effect of *APOE*4 genotype on the cellular senescence in the aging brain. Through the metabolomic and transcriptomic means, we found major changed metabolites in the brain of aging ApoE4 mice. We also showed that the administration of key metabolic intermediates rescued the cellular senescence and improved cognitive function in the aging ApoE4 mice. These findings may explain the mechanisms underlying the ApoE4‐increased LOAD risk and provide potential therapeutic targets for the cognitive improvement of *APOE*4 carriers.

## 2 MATERIALS AND METHODS

### 2.1 Animals and drug treatments

C57BL/6J mice with human *APOE* (E3/E4) target replacement (TR) were obtained from Taconic Biosciences (Rensselaer, NY, USA), in which mouse *APOE* was replaced with human *APOE*. The homozygous genetic background was confirmed as previously described (Levi et al., 2003). The animals were bred in Fujian Medical University and the animal‐related study protocol was approved and followed the rules and regulations by Animal Care and Use Committee of Fujian Medical University (IACUC FJMU 2021–0272). We chose female ApoE3‐ and ApoE4‐TR mice for our research. Those mice were randomized into vehicle/drug treatment group before experiments. Glycerol Triacetate (GTA) (Cat. 90240; Sigma, USA), an FDA‐approved additive, was obtained and dissolved in ddH_2_O containing 0.5% carboxymethyl cellulose sodium and 2% Tween‐80. GTA (2 g/Kg/day) was administered to those mice by gavage every day for 40 days.

### 2.2 Morris water maze test

After GTA treatment, Morris water maze was conducted as previously reported (Zhang et al., 2019). Briefly, mice received four 1‐min trainings per day in a 40 cm × 40 cm pool from four different directions in a randomized order for 5 days. A hidden platform was placed in a designated location for the mice to rest. After the five‐day trainings, the hidden platform was removed and each mouse was placed into the pool from a totally different direction. A detailed record was made for each mouse regarding the number of crossings over the hidden platform, time in the targeted quadrant, first escape time, and swimming speed.

### 2.3 Tissue preparation

All animals were sacrificed after the Morris water maze test. Briefly, the animals were deeply anesthetized with isoflurane and rapidly perfused with ice‐cold 0.01 M PBS (about 20 mL each) from the left ventricle to evacuate blood from the brain. Hippocampus and cortex were immediately separated on ice and followed by freezing in liquid nitrogen. Tissues were subsequently frozen in a −80°C refrigerator for further experiments. For IHC or β‐Gal staining, freshly‐removed cerebrums were fixed in 4% paraformaldehyde at 4°C for 24 h and then transferred to 30% sucrose at 4°C for 7 days before slicing.

### 2.4 Synaptic protein extraction

Synaptic protein extraction was performed with Syn‐PER Synaptic Protein Extraction Reagent (87793; Thermo Scientific, USA) as described previously (Zhang et al., 2019). Briefly, the samples were weighed and moved into the Syn‐PER Reagent containing 1% protease inhibitor cocktail (Roche) to achieve homogeneity. After the first centrifugation (12,000 *g*, 10 min), the supernatant was removed to another tube for a second centrifugation (15,000 *g*, 20 min) to collect synaptosome pellet. Afterwards, the synaptosome pellet was resuspended in the Syn‐PER Reagent for further experiments.

### 2.5 Western blot analysis

Western blotting was performed as described previously (Liao et al., 2021; Zhang et al., 2019). Briefly, each tissue was lysed in a RIPA buffer (50 mM Tris–HCl pH 8.0, 150 mM NaCl, 1% NP40, 0.5% sodium‐deoxycholate, and 0.1% SDS) containing protease inhibitor cocktail (Cat# P8340; Sigma, USA) and phosphatase inhibitors (20 mM Na_4_P_2_O_7_, 1 mM NaF, and 1 mM Na_3_VO_4_), sonicated for 1 min, and centrifuged at 12,000 rcf for 25 min. After extraction, the supernatant was obtained and the concentration of the protein solution was detected with a BCA protein assay kit (Cat# P0009; Beyotime, China). The protein concentration in each tube was equalized before a 10‐min boiling.

Subsequently, the samples were loaded and separated with 8%–12% sodium dodecyl sulfate polyacrylamide gel electrophoresis (SDS‐PAGE) and immediately transferred to the polyvinylidene fluoride (PVDF) membrane at 110 V for 90 min. Afterwards, the membrane was blocked for 1 h with Tris‐buffered saline Tween‐20 (TBST, pH 7.6; containing 10 mM Tris, 150 mM NaCl, and 0.1%Tween‐20) containing 5% bovine serum albumin (BSA) and incubated overnight with primary antibodies that were solved in TBST containing 2.5% BSA. Then, the membrane was washed three times (10 min/time) and incubated with secondary antibodies conjugated with horseradish peroxidase in TBST. After another three washes, the e ELU Chemiluminescent Substrate System was applied to the membrane before exposure. Densitometric analysis was performed with the NIH Image J software.

The antibodies used were as follows: mTOR (1:1000, #2983; CST, USA), phosphorylated‐mTOR (1:1000, #5536; CST, USA), VGLUT1 (1:1000, ab134283; Abcam, UK), synaptophysin (1:2000, ab32127; Abcam, UK), PSD95 (1:1000, ab18258; Abcam, UK), NR2B (1:1000, ab254356; Abcam, UK), NR2A (1:1000, ab124913; Abcam, UK), β‐actin (1:1000, ab8226; Abcam, UK), Goat Anti‐Rabbit IgG H&L (HRP) (1:10000, ab205718; Abcam, UK), Goat Anti‐Mouse IgG H&L (HRP) (1:10000, ab205719; Abcam, UK).

### 2.6 RNA extraction and real‐time quantitative PCR

Real‐time quantitative PCR (RT‐qPCR) was performed as described previously (Zhang et al., 2019). In this study, the RNA was extracted from tissues with the Trizol reagent according to manufacturer's instructions (Invitrogen, USA). The reverse transcription was conducted with a Revert Aid First Strand cDNA Syn thesis Kit (Thermo, USA) with the concentration of each sample equalized to 1μg/ul in a 20‐μl volume. The fluorescence was measured using the Step‐One Plus real‐time PCR system (Life Technologies Applied Biosystems, Grand Island, NY), with the number of cycles calculated with the 2^−∆∆CT^ method. The sequences used in this study were detailed in Table S1.

### 2.7 Immunohistochemical and senescence‐associated β‐galactosidase (SA‐β‐gal) staining

All tissue slides were sliced with a microtome (CM1850; Leica, Germany) at 40 μm and subjected to immunohistochemical and SA‐β‐gal stainings. The SA‐β‐gal staining was conducted with a β‐Gal staining Kit (C0605; Beyotime, China) and slides were incubated with a mixture of reagents A, B, C, and X‐Gal at 37°C overnight. The slides were observed under a bright light microscope after the TBST wash.

Immunohistochemical staining was conducted, as previously described (Zhang et al., 2014), with a UltraSensitive™ SP IHC Kit (KIT‐9720; MXB biotechnologies, China). Floating tissue slides were washed in TBS for three times. Then, reagent A and B from the kit were used before the incubation with primary antibodies at 4°C overnight. Afterwards, reagent C and D were applied before staining slides with DAB kit (MAX‐002; MXB biotechnologies, China).

The antibodies used in immunochemical staining were as follows: NeuN (1:1000, ab177487; Abcam, UK), GFAP (1:1000, ab7260; Abcam, UK), and Iba‐1 (1:2000, 016–20,001; Wako, Japan).

### 2.8 Ac‐CoA detection

Ac‐CoA detection was performed as described previously (Huang et al., 2021). In brief, the level of Ac‐CoA in brain tissues was detected with a Mouse Ac‐CoA ELISA Kit (EY12009‐M; Shanghai Yiyan Bio‐technology Co. Ltd, China). The absorbance was determined at 450 nm by referring to the standard curve.

### 2.9 Measurement of activities of mitochondrial complex and citroyl synthetase

Isolation of mitochondrial complex was performed as described in a previous study (Rhein et al., 2009). After isolation, the activities of mitochondrial complex were measured with amitochondrial complex I kit (YIJI, Shanghai). Cytochrome C oxidase was evaluated with a Cytochrome C Oxidase Assay Kit (Cat. CYTOCOX1; Sigma, USA). Citroyl synthetase was measured with a Citrate Synthase Assay Kit (Cat. CS0720; Sigma, USA). The activity of mitochondrial complex was detected at 340 nm and 380 nm with a spectrophotometer. A negative control was conducted to ascertain the obtained data. Citric acid synthase activity was measured at 412 nm during the first and fifth minutes.

### 2.10 Measurement of ATP level and NAD/NADH ratio

ATP level was measured as previously published (Fang et al., 2021). In brief, the extracted supernatant was collected after sonication in a lysis buffer (1 M Na_3_VO_4_, 2 mM NaF, 2.5 mM Na_4_P_2_O_7_, 1% Triton X‐100, and 1% protease inhibitor cocktail) and centrifugation (12,000 *g*, 25 min, 4°C). Then, protein concentration was identified with a BCA kit and ATP was with an ATP Bioluminescence Assay Kit HS II (Roche Molecular Biochemicals, Germany). The ratio of NAD/NADH was calculated as previously described (Reyes et al., 2008). NADH (Cat. N4505; Sigma, USA) was used and incubated with an equal number of isolated mitochondria and the NAD absorbance was monitored at 340 nm.

### 2.11 Untargeted metabolism of hippocampus

The ultrahigh performance liquid tandem chromatography quadrupole time‐of‐flight mass spectrometry (UHPLC‐QTOFMS) method was applied to complete untargeted metabolic profiling in Shanghai Biotree Biotech Co., Ltd., following the previous protocols (Y. Zhao et al., 2019). Briefly, tissues were homogenized in ddH_2_O and Waters ACQUITY UPLC BEH C18 column (2.1 mm × 100 mm; 1.7 μm) with triple time‐of‐flight 6600 (Q‐TOF; AB Sciex, Framingham, MA, USA). The mobile phase of A (containing 0.1% formic acid in water) was used as positive mode and ammonium acetate (5 mmol/L) in water as negative mode. Acetonitrile was chosen as mobile phase B. The steps of elution were conducted at a flow rate of 500 μl/min as follows: 0–1.0 min, 1% B; 1.0–8.0 min, 1–99% B; 8.0–10.0 min, 99% B; 10.0–10.1 min, 99% ~ 1% B; and 10.1–12 min, 1% B. The continuous full scan MS spectrum was monitored with Thermo Xcalibur 4.0.27 coupled with QE mass spectrometer on the information‐dependent acquisition (IDA) mode. Then, ESI source conditions were set as described previously (Cai et al., 2021). All data were analyzed on the BGISEQ‐500 platform.

### 2.12 Bulk RNA‐sequencing

Bulk RNA‐Sequencing of whole hippocampus was conducted in Beijing novogene Biotech Co., Ltd. Briefly, after extraction of RNA and quality integrity test, cDNA was reversed from purified RNA. All genes involved in the library were aligned by using HISAT2. According to mouse reference genome Ensembl_release104, we identified expression abundance and variations for each of the genes and normalized them to fragments per kilobase of transcript per million mapped reads (FPKM) using RNA‐seq by Expectation Maximization (RSEM). The differentially expressed genes (DEGs) in different groups were identified by the standard of a fold change ≥1 and adjusted *p* values <0.05. All DEGs were clustered and analyzed in Kyoto Encyclopedia of Genes and Genomes (KEGG) database.

### 2.13 Transmission electronic microscopy

Transmission electronic microscopy was performed as described previously (Gu et al., 2018). Briefly, after the tissue collection, samples were fixed with a fixation buffer (3% glutaraldehyde, 1.5% paraformaldehyde in 0.1 M PBS) for serval days. Then dehydration, embedding and polymerization of the samples were performed. Subsequently, the prepared samples were sliced into sections (about 15 μm each) with a slicer (LeicaUC‐6) and observed by transmission electronic microscopy (PHILIPSEM208). Finally, the number of synapses in each figure of each mouse was counted and averaged and length (nm) and thickness (nm) of PSD of each synapse were measured with ImageJ.

### 2.14 Statistical analysis

All data were processed with Graphpad Prism 6.0 and presented as mean ± sem value. All data were analyzed by appropriate statistical methods in different experiments. All data between groups were analyzed by two‐tailed Student's *t*‐tests and those among four groups by two‐way ANOVA. The escape latency in the Morris water maze test was evaluated by ANOVA for repeated measurement. The details were indicated in different figure legends. *p*<0.05 was considered as statistically significant.

## 3 RESULTS

### 3.1 *APOE* ε4 allele accelerates the senescence of hippocampal neurons in an age‐dependent manner

**FIGURE 1:**
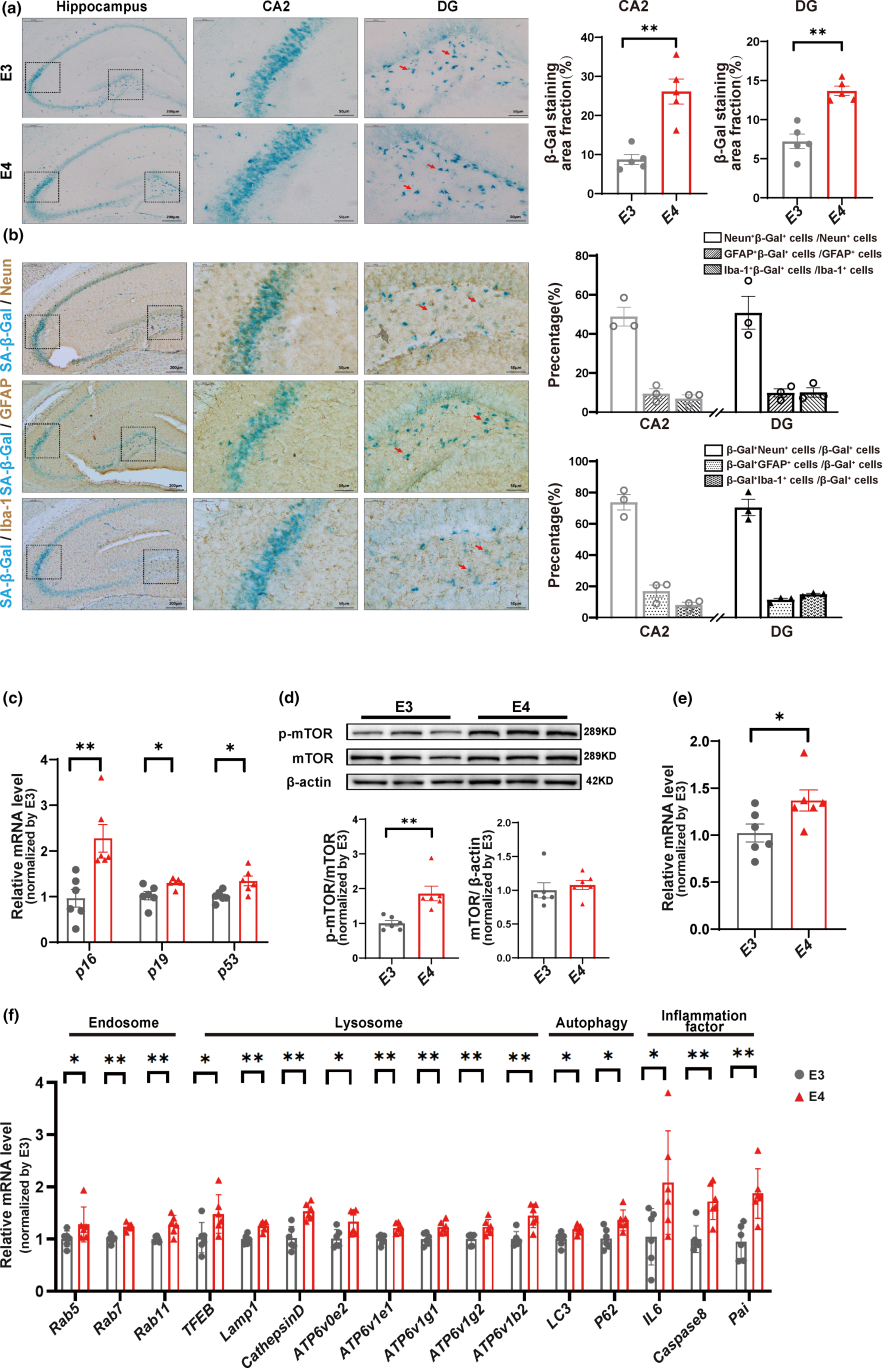
The accumulation of senescent neurons in the hippocampus of the elderly ApoE4‐TR mice. (a) Representative images (left) and quantitative analyses (right) of SA‐β‐Gal staining in the hippocampus of 18‐month‐old ApoE3‐ and ApoE4‐TR mice. The red arrow indicates those senescent cells in CA2 and DG. Scale bars: 200 μm and 50 μm, respectively. CA2 E3 versus E4, *F* = 44.54 *p* = 0.0001; DG E3 versus E4, *F* = 2.015 *p* < 0.0001; *n* = 5 mice per group. (b) Representative images (left) and quantitative analyses (right) of brain sections costained with NeuN, GFAP, Iba1, and SA‐β‐gal to label Neurons, Astrocytes, Microglia (brown) and Senescent cells (blue), and the merged. Scale bars: 200 μm and 50 μm, respectively. *n* = 3 mice per group. (c) Quantified expressions of P16, P19, and P53 in the hippocampus by real‐time PCR. P16 E3 versus E4 *F* = 2.377 *p* = 0.0044; P19 E3 versus E4 *F* = 4.801 *p* = 0.0184; P53 *F* = 4.429 *p* = 0.0161. *n* = 6 mice per group. (d, e) Western blot analysis and quantitative real‐time PCR analysis of p‐mTOR and mTOR in the hippocampus. WB mTOR/β‐actin E3 versus E4 *F* = 2.691 *p* = 0.5585; p‐mTOR/mTOR *F* = 7.630 *p* = 0.0035; qPCR E3 versus E4 *F* = 1.391 *p* = 0.0405. *n* = 6 mice per group. (f) Quantified expressions of endosome genes (Rab5, Rab7, and Rab11), lysosome genes (TFEB, Lamp1, Cathepsin D, ATP6v0e2, ATP6v1e1, ATP6v1g1, Atp6v1g2, and Atp6v1b2), autophagy genes (LC3 and P62), and pro‐inflammatory factors (IL‐6, caspase8, and Pai) in the hippocampus. Rab5 E3 versus E4 *F* = 4.529 *p* = 0.0335; Rab7 E3 versus E4 *F* = 1.136 *p* = 0.0001; Rab11 E3 versus E4 *F* = 11.51 *p* = 0.0047; TFEB E3 versus E4 *F* = 1.623 *p* = 0.0401; Lamp1 E3 versus E4 *F* = 1.107 *p* = 0.0006; Cathepsin D E3 versus E4 *F* = 2.011 *p* = 0.001; ATP6v0e2 E3 versus E4 *F* = 1.787 *p* = 0.0207; ATP6v1e1 E3 versus E4 *F* = 1.787 *p* = 0.0207; ATP6v1g1 E3 versus E4 *F* = 1.102 *p* = 0.0057; ATP6v1g2 E3 versus E4 *F* = 2.573 *p* = 0.0023; LC3 E3 versus E4 *F* = 2.439 *p* = 0.0156; P62 E3 versus E4 *F* = 1.038 *p* = 0.0116; IL6 E3 versus E4 *F* = 3.1228 *p* = 0.0377; Caspase8 E3 versus E4 *F* = 1.828 *p* = 0.0021; Pai E3 versus E4 *F* = 2.137 *p* = 0.0029. *n* = 6 mice per group. Data are expressed as Mean ± SEM. *p < 0.05 **p < 0.01, by two‐tailed Student's *t*‐tests.

Although the ε4 allele of *APOE* has been documented as the strongest genetic risk factor for AD when compared with the common ε3 allele and the protective ε2 allele (Castellano et al., 2011; Reiman et al., 2009), the effect of ApoE4 on the neuronal senescence in the brain remains unclear. Therefore, we examined the aging‐related changes in the hippocampus of ApoE3‐ and ApoE4‐TR mice at 9 and 18 months of age, indicating the mid‐aged and elderly mice, respectively. Intriguingly, we found that compared with the age‐matched ApoE3‐TR counterparts, the 18‐month‐old ApoE4‐TR mice reported a concentration of SA‐β‐gal‐positive cells in the CA2 and dentate gyrus (DG) areas and a stronger SA‐β‐gal staining intensity in the hippocampus (Figure 1a). Combined with immunohistochemical staining, the SA‐β‐gal staining was more colocalized with neuron makers (NeuN), but less colocalized with astrocytes (GFAP) or microglia (IBA1), indicating senescence occurs primarily in neurons rather than in astrocytes or microglia in the hippocampus of elderly ApoE mice (Figure 1b). To further explore the extent of aging in the hippocampus of ApoE‐TR mice, the mRNA levels of classical senescent markers (P16, P19, and P53) were quantified, which revealed significantly higher expressions of P16, P19, and P53 in the elderly ApoE4‐TR mice than in the age‐matched E3 mice (Figure 1c). These phenomena were not significantly different in the 9‐month ApoE3/E4‐TR mice (Figure S1a).

Accumulative evidence suggests that the mTOR activation plays a key role in maintaining the survival of senescent cells and promoting the senescence‐associated secretory phenotype (SASP) (Carroll et al., 2017; Herranz et al., 2015; Xu et al., 2014). In most cases, mTOR regulates protein synthesis and autophagic degradation in an opposite manner; however, mTOR and autolysosomes can accumulate in the Golgi apparatus to handle rapid protein turnover and promote SASP during Ras‐induced senescence (Narita et al., 2011). Therefore, we explored the changes of senescence‐related mTOR signaling and endolysosomal‐autophagic (ELA) system in the hippocampus of these mice. As expected, compared with the age‐matched ApoE3‐TR mice, a significantly increased ratio of p‐mTOR/mTOR was detected in the hippocampus of the 18‐month‐old ApoE4‐TR mice (Figure 1d,e) while no marked difference was evident in the 9‐month‐old ApoE3‐ and ApoE4‐TR mice (Figure S1b,c). Furthermore, compared with the 18‐month‐old ApoE3‐TR mice, the age‐matched ApoE4‐TR mice reported obviously higher mRNA levels of endosome markers (Rab5, Rab7, and Rab11), lysosomal markers (TFEB, Lamp1, Cathepsin D, ATP‐1 g1, and ATP‐1 g2), autophagy markers (LC3 and p62), and inflammation makers (IL6, Caspase 8, and Pai) (Figure 1f) while no significant difference was found in the 9‐month‐old ApoE3‐ and ApoE4‐TR mice (Figure S1d).

Collectively, these findings suggest that ApoE4 accelerates the senescence of hippocampal neurons, which is accompanied with the activation of mTOR and ELA system in an age‐dependent manner.

### 3.2 APOE ε4 alters the metabolic and transcriptomic profile of the hippocampus in an age‐dependent manner

**FIGURE 2:**
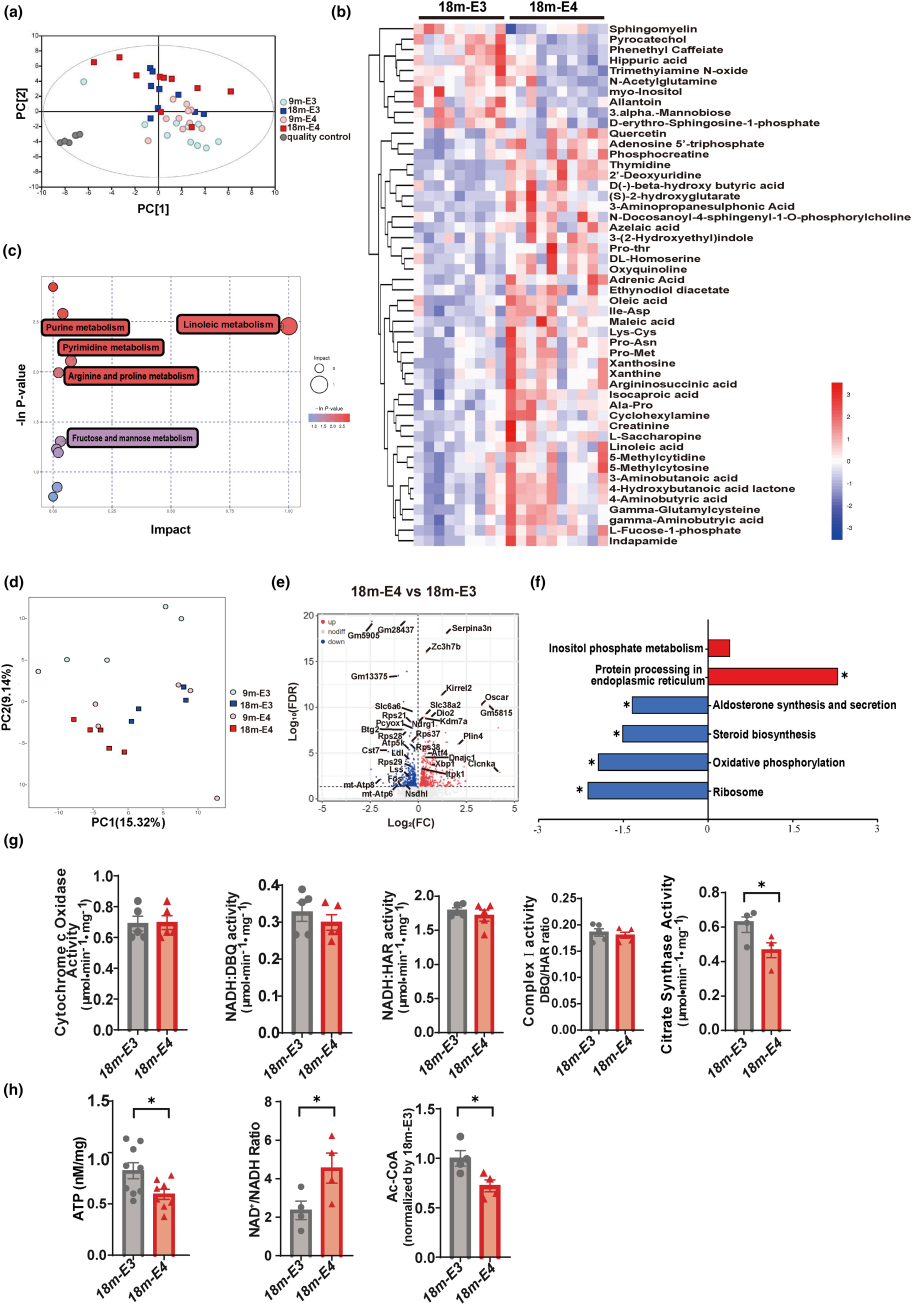
The decline of Ac‐CoA in the hippocampus of 18‐month‐old ApoE4‐TR mice by metabolic and transcriptomic profiling combined with biochemical analysis. (a–c) PCA analysis for untargeted metabolomic profiling (a). Heat map for differential metabolites in the hippocampus between 18‐month‐old ApoE3 and ApoE4 mice (b). KEGG signaling pathway analysis for differential metabolites (c). *n* = 10 mice per group. (d–f) PCA analysis for bulk RNA‐sequencing in the hippocampus (d). Volcanic map (e) and KEGG signaling pathway (f) analysis for differential genes between 18‐month‐old ApoE3 and ApoE4 mice. *n* = 4–5 mice per group. (g) Quantification of the activation of mitochondrial complex and citrate synthase in the hippocampus of 18‐month‐old ApoE3 and ApoE4 mice. *n* = 5 mice per group. Cytochrome c Oxidase Activity E3 versus E4 *F* = 1.12 *p* = 0.9218; NADH: DBQ activity E3 versus E4 *F* = 1.498 *p* = 0.4226; NADH: HAR activity E3 versus E4 *F* = 4.532 *p* = 0.4090; complex I activity E3 versus E4 *F* = 1.679 *p* = 0.5756; Citrate synthase activity *F* = 1.026 *p* = 0.036. (h) Quantification of the levels of ATP, NAD+, and Ac‐CoA in the hippocampus of 18‐month‐old ApoE3 and ApoE4 mice. *n* = 4–9 mice per group. ATP E3 versus E4 *F* = 2.822 *p* = 0.0284; NAD+/NADH E3 versus E4 *F* = 6.313 *p* = 0.0442; Ac‐coA E3 versus E4 *F* = 1.7 *p* = 0.0313. Data are expressed as Mean ± SEM. *p < 0.05, by two‐tailed Student's *t*‐tests.

To investigate the age‐dependent effects of ApoE4 on the hippocampus, we assessed the metabolic and transcriptomic profile in the hippocampal tissues of ApoE3‐ and ApoE4‐TR mice at 9 and 18 months of age. The untargeted metabolomic results reported a total of 3141 metabolites within the hippocampi of ApoE3 and E4 mice, which were further subjected to the principal component analysis (PCA) for a global metabolomic profiling. The analysis revealed that the overall profile of ApoE4 mice shifted from that of ApoE3 mice in an age‐dependent manner (Figure 2a). With the standard set at VIP >1 and *p* < 0.05, 50 of the identified 204 differentially‐expressed metabolites were further mapped for 18‐month‐old ApoE3‐ and ApoE4‐TR mice. Heat maps of the hierarchical clustering of differentially‐expressed metabolites showed distinct patterns of change between 18‐month‐old ApoE3 and ApoE4 mice. Among these metabolites, substances related to lipid metabolic pathways were most highly enriched, including sphingomyelin (SM), D‐erythro‐sphingosine‐1‐phosphate (S1P), N‐docosanoyl‐4‐sphingenyl‐1‐O‐ phosphorylcholine, beta‐hydroxybutyric acid, ethynodil diacetate, oleic acid, and linoleic acid (Figure 2b,c). The same procedure was performed for the differentially‐expressed metabolites in 9‐month‐old ApoE3 and ApoE4 mice, which, except for S1P and 1‐stearoyl‐2‐oleoyl‐sn‐glycerol 3‐phosphocholine (SOPC), reported an enrichment of D‐Glucose 6‐phosphate, beta‐D‐fructose 6‐phosphate, acetyl‐phosphate, ADP‐ribose, D‐erythrose 4‐phosphate, and 2^’^‐deoxy‐D‐ribose (Figure S2a,b).

To further confirm the age‐dependent effects of ApoE4 on the hippocampus, an unbiased approach was adopted for the analysis of the entire transcriptome in the contralateral hippocampal tissue of each mouse using a BGISEQ‐500 platform. The PCA showed a clear distinction in the transcriptomic variation between the ApoE3 and ApoE4 groups, regardless of their ages (Figure. 2d). Compared with the age‐matched ApoE3 mice, the 18‐month‐old ApoE4 mice reported 589 upregulated genes and 425 downregulated genes (adjusted *p* value <0.05, fold change≥1). Notably, endoplasmic reticulum stress genes including Atf4 and Xbp1, mitochondrial complex related genes including mt‐ATP8, mt‐ATP6, and Atp5k, lipid droplet formation related gene Plin4, oxidative damage maker Serpina3n, were significantly difference between ApoE4 mice and ApoE3 mice (Figure. 2e). The KEGG analysis revealed that the downregulated pathways were associated with oxidative phosphorylation (OP), steroid biosynthesis, and aldosterone synthesis and secretion, while the upregulated pathways were closely linked with protein processing in the endoplasmic reticulum (Figure 2f). The same procedure was performed to analyze the differentially‐expressed genes between 9‐month‐old ApoE3 and ApoE4 mice, revealing that Serpina3n and Atf4 were increased in ApoE4 mice compared with ApoE3 mice. Moreover, the most significantly upregulated pathway by ApoE4 was aminoacyl‐tRNA biosynthesis (Figure S2c,d).

Together, these results suggest that ApoE4 affects the metabolic and transcriptomic profile of the hippocampus in an age‐dependent manner, with more prominent changes in lipid metabolism in an old age and pronounced changes in glucose metabolism in a middle age.

### 3.3 Acetyl‐CoA level significantly decreases in the hippocampus of the elderly ApoE4 mice

As a derivative from tricarboxylic acid cycle (TCA) in mitochondria, Ac‐CoA is one of the main sources of lipid synthesis. In light of the above metabolic and transcriptomic data, we further examined the indicators related to the mitochondrial function. Surprisingly, the activity of citrate synthase in the hippocampal mitochondrial extracts significantly decreased in the 18‐month‐old ApoE4 mice when compared with that in the ApoE3 counterparts, while no difference was found in the activities of complex I and cytochrome c oxidase (complex IV) (Figure 2g). As expected, in the hippocampal homogenates of the 18‐month‐old ApoE4 mice, the ATP level obviously decreased; the ratio of NAD^+^/NADH increased; and, most importantly, the level of total Ac‐CoA was significantly reduced, indicating a marked decrease in the citrate synthase activity in the mitochondria of the 18‐month‐old ApoE4 mice (Figure 2h). However, these changes were not observed in 9‐month‐old ApoE4 mice (Figure S2e,f). The above results indicate that ApoE4 may accelerate the decrease of hippocampal Ac‐CoA in an age‐dependent manner.

### 3.4 Ac‐CoA supplementation rescues the hippocampal senescence of the elderly ApoE4 mice

**FIGURE 3:**
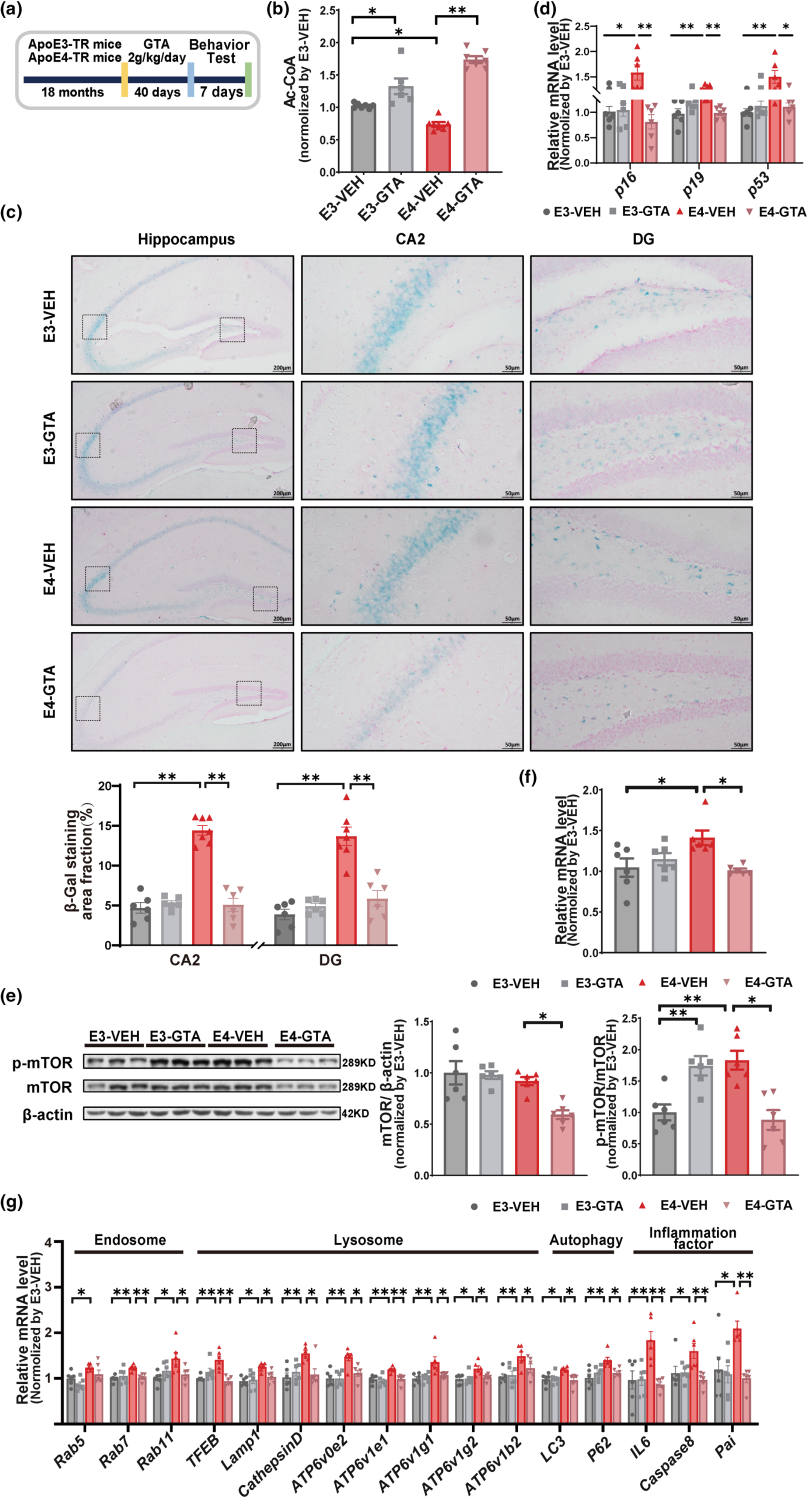
The rescued senescence in the hippocampus of 18‐month old ApoE4‐TR mice by GTA supplementation. (a) Schematic diagram depicts the GTA treatment in the 18‐month‐old ApoE3‐ and ApoE4‐ TR mice. (b) Quantification of the level of Ac‐CoA in the hippocampus using ELISA kit. *F* = 8.05 E3‐VEH versus E4‐VEH *p* = 0.0159; E3‐VEH versus E3‐GTA *p* = 0.0141; E4‐VEH versus E4‐GTA *p* < 0.0001; *n* = 6–7 mice per group. (c) Representative images (up) and quantitative analyses (down) of SA‐β‐Gal staining in the hippocampus of 18‐month‐old ApoE3‐ and ApoE4‐TR mice treated by GTA. CA2 *F* = 60.61 E3‐VEH versus E4‐VEH *p* < 0.0001; E4‐VEH versus E4‐GTA *p* < 0.0001; DG *F* = 27.72 E3‐VEH versus E4‐VEH *p* < 0.0001 E4‐VEH versus E4‐GTA *p* < 0.0001; *n* = 6–7 mice per group. (d) Quantification of the expressions of P16, P19, and P53 in the hippocampus. P16 *F* = 6.494 E3‐VEH versus E4‐VEH *p* = 0.0262 E4‐VEH versus E4‐GTA *p* = 0.0022; P19 *F* = 7.5 E3‐VEH versus E4‐VEH *p* = 0.0031 E4‐VEH versus E4‐GTA *p* = 0.0049; P53 *F* = 5.702 E3‐VEH versus E4‐VEH *p* = 0.0047 E4‐VEH versus E4‐GTA *p* = 0.0326; *n* = 6 mice per group. (e, f) Western blot analysis and quantitative real‐time PCR analysis of p‐mTOR and mTOR in the hippocampus. WB mTOR/β‐actin *F* = 8.188 E3‐VEH versus E4‐VEH *p* = 0.8229 E4‐VEH versus E4‐GTA *p* = 0.0117; p‐mTOR/mTOR *F* = 11.18 E3‐VEH versus E4‐VEH *p* = 0.0037 E4‐VEH versus E4‐GTA *p* = 0.001. qPCR *F* = 4.002 E3‐VEH versus E4‐VEH *p* = 0.0339 E4‐VEH versus E4‐GTA *p* = 0.0327; *n* = 6 mice per group. (g) Quantification of the expressions of endosome genes (Rab5, Rab7, and Rab11), lysosome genes (TFEB, Lamp1, Cathepsin D, ATP6v0e2, ATP6v1e1, ATP6v1g1, Atp6v1g2, and Atp6v1b2), autophagy genes (LC3 and P62), and pro‐inflammatory factors (IL‐6, caspase8, and Pai) in the hippocampus. Rab5 *F* = 6.405 E3‐VEH versus E4‐VEH *p* = 0.0211 E4‐VEH versus E4‐GTA *p* = 0.0609; Rab7 *F* = 6.669 E3‐VEH versus E4‐VEH *p* = 0.0033 E4‐VEH versus E4‐GTA *p* = 0.0081; Rab11 *F* = 4.624 E3‐VEH versus E4‐VEH *p* = 0.0115 E4‐VEH versus E4‐GTA *p* = 0.0426; TFEB *F* = 10.63 E3‐VEH versus E4‐VEH *p* = 0.0011 E4‐VEH versus E4‐GTA *p* = 0.0003; Lamp1 *F* = 5.502 E3‐VEH versus E4‐VEH *p* = 0.0202, E4‐VEH versus E4‐GTA *p* = 0.0474; Cathepsin D *F* = 6.102 E3‐VEH versus E4‐VEH *p* = 0.005 E4‐VEH versus E4‐GTA *p* = 0.0137; ATP6v0e2 *F* = 9.783 E3‐VEH versus E4‐VEH *p* = 0.0008 E4‐VEH versus E4‐GTA *p* = 0.0108; ATP6v1e1 *F* = 1.294 E3‐VEH versus E4‐VEH *p* = 0.004 E4‐VEH versus E4‐GTA *p* = 0.005; ATP6v1g1 *F* = 6.178 E3‐VEH versus E4‐VEH *p* = 0.0060 E4‐VEH versus E4‐GTA *p* = 0.0344; ATP6v1g2 *F* = 5.115 E3‐VEH versus E4‐VEH *p* = 0.0148 E4‐VEH versus E4‐GTA *p* = 0.0457; ATP6v1b2 *F* = 6.557 E3‐VEH versus E4‐VEH *p* = 0.0043 E4‐VEH versus E4‐GTA *p* = 0.0230; LC3 *F* = 4.94 E3‐VEH versus E4‐VEH *p* = 0.0282 E4‐VEH versus E4‐GTA *p* = 0.0148; P62 *F* = 6.672 E3‐VEH versus E4‐VEH *p* = 0.0019 E4‐VEH versus E4‐GTA *p* = 0.0279; IL6 *F* = 8.596 E3‐VEH versus E4‐VEH *p* = 0.0037 E4‐VEH versus E4‐GTA *p* = 0.0013; Caspase 8 *F* = 5.051 E3‐VEH versus E4‐VEH *p* = 0.0488 E4‐VEH versus E4‐GTA *p* = 0.0077; Pai *F* = 7.053 E3‐VEH versus E4‐VEH *p* = 0.0163 E4‐VEH versus E4‐GTA *p* = 0.0031; *n* = 6 mice per group. Data are expressed as mean ± SEM. * *p* < 0.05 ** *p* < 0.01, calculated by two‐way ANOVA.

To investigate whether Ac‐CoA reduction is the culprit for the aging of hippocampus in ApoE4‐TR mice, ApoE‐TR mice at 18 months of age received by gavage a GTA administration (Figure 3a), an FDA‐approved food additive that rapidly increases the Ac‐CoA level in the brain (Mathew et al., 2005; Reisenauer et al., 2011). As expected, GTA administration (2 g/kg/day for 40 days) significantly increased the level of Ac‐CoA (Figure 3b) and reduced the intensity of SA‐β‐Gal staining (Figure 3c) in the hippocampus of 18‐month‐old ApoE4 mice. Surprisingly, the mRNA levels of classical senescent markers (P16, P19, and P53) were significantly decreased in the ApoE4‐GTA mice compared with ApoE4‐VEH counterparts (Figure 3d). Meanwhile, GTA administration increased the phosphorylation of mTOR in the hippocampus of 18‐month‐old ApoE3 mice, consistent with the previous findings that intracellular energy signals (amino acids, glucose, and oxygen, etc.) stimulate mTORC1 activity (Dibble & Manning, 2013; Son et al., 2019) (Figure 3e). However, the same GTA treatment in the 18‐month‐old ApoE4 mice downregulated the mTOR signaling (Figure 3e,f) and the levels of endosomes markers (Rab7, Rab11), lysosomal markers (Lamp1, Cathepsin D, ATP6v0e2, ATP6v1e1, ATP6v1g1, ATP6v1g2, and ATP6v1b2), autophagy markers (LC3, p62), and inflammatory factors (IL‐6, Caspase 8, and Pai) (Figure 3g). The above results suggest that Ac‐CoA supplementation may rescue the aggravated hippocampal senescence of the elderly ApoE4 mice.

### 3.5 Ac‐CoA supplementation enhances the synaptic structure and spatial memory of the elderly ApoE4 mice

**FIGURE 4:**
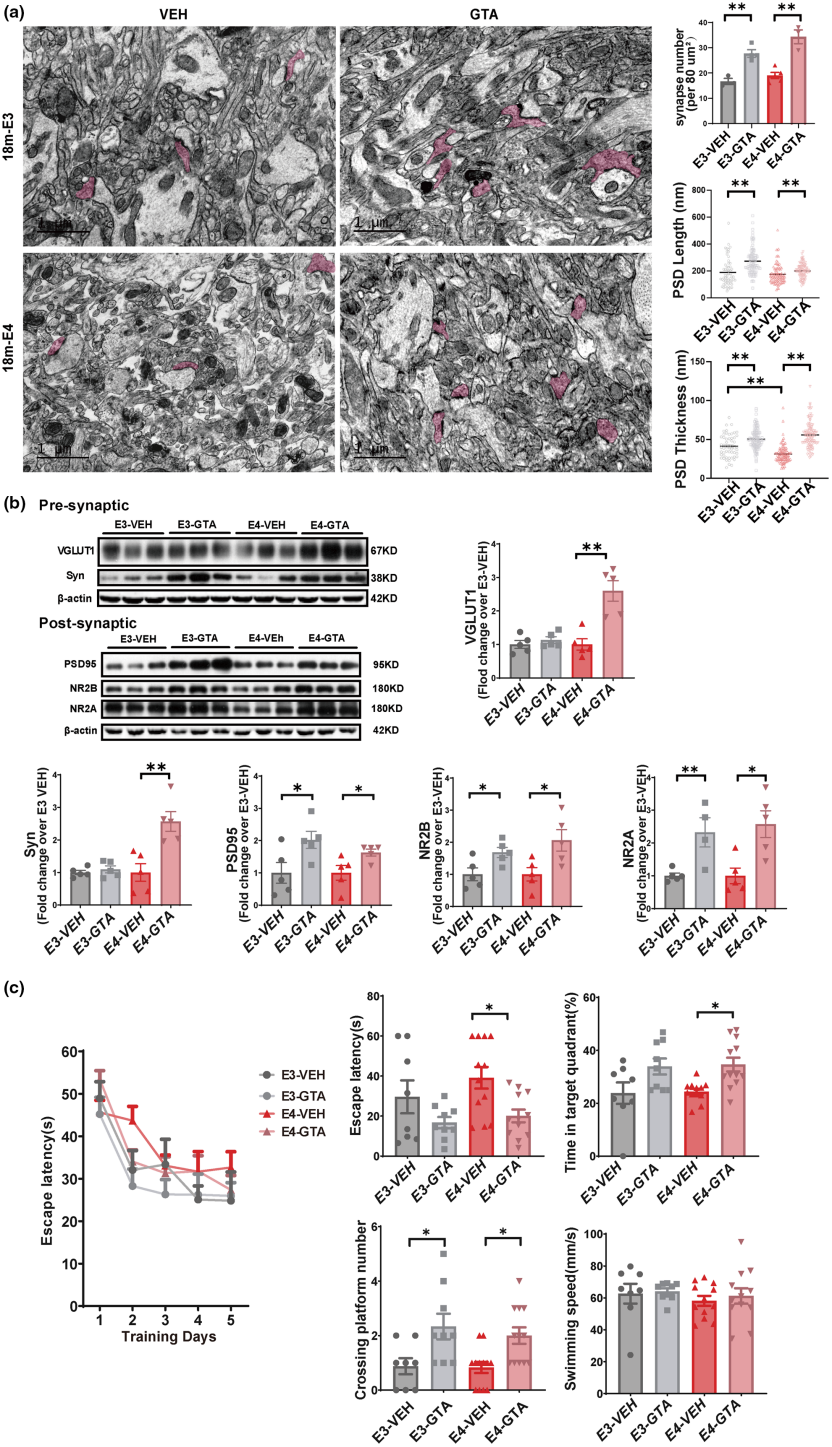
The enhanced synaptic plasticity and spatial memory in the 18‐month‐old ApoE4‐TR mice by GTA supplementation. (a) Representative TEM images (left) and quantitative analyses (right) of the typical synaptic structure of hippocampi CA1. Red areas indicate for presynaptic regions. Scale bar, 1 μm. *n* = 3, 4, 5, 3 mice for ApoE3‐VEH, ApoE3‐GTA, ApoE4‐VEH, and ApoE4‐GTA, respectively. Synapse number *F* = 12.08, E3‐VEH versus E4‐VEH *p* = 0.7471; E3‐VEH versus E3‐GTA *p* = 0.0037; E4‐VEH versus E4‐GTA *p* = 0.0002; PSD length *F* = 3.41 E3‐VEH versus E4‐VEH *p* = 0.0002; E3‐VEH versus E3‐GTA *p* = 0.0006; E4‐VEH versus E4‐GTA *p* < 0.0001. PSD thickness *F* = 5.14 E3‐VEH versus E4‐VEH *p* > 0.9999; E3‐VEH versus E3‐GTA *p* < 0.0001; E4‐VEH versus E4‐GTA *p* = 0.0090. (b) Representative immunoblots and Western blot analysis of synaptic plasticity‐associated proteins in the hippocampal synaptosome extract, *n* = 5 mice per group. VGLUT1 *F* = 1.424 E3‐VEH versus E4‐VEH *p* > 0.9999, E3‐VEH versus E3‐GTA *p* = 0.4134, E4‐VEH versus E4‐GTA *p* = 0.0018; Syn *F* = 1.246 E3‐VEH versus E4‐VEH *p* > 0.9999, E3‐VEH versus E3‐GTA *p* = 0.4590, E4‐VEH versus E4‐GTA *p* = 0.0046; PSD *F* = 1.990 E3‐VEH versus E4‐VEH *p* > 0.9999, E3‐VEH versus E3‐GTA *p* = 0.0396, E4‐VEH versus E4‐GTA *p* = 0.0361; NR2B *F* = 1.080 E3‐VEH versus E4‐VEH *p* > 0.9999, E3‐VEH versus E3‐GTA *p* = 0.0282, E4‐VEH versus E4‐GTA *p* = 0.0278; NR2A *F* = 8.491 E3‐VEH versus E4‐VEH *p* > 0.9999 E3‐VEH versus E3‐GTA *p* = 0.0058 E4‐VEH versus E4‐GTA *p* = 0.0103. (c) GTA‐treated mice were tested in the Morris water maze. Escape latency to the platform position during the training trials (1–5d) (5th day, *F* = 26.02, E3‐VEH versus E4‐VEH *p* = 0.7431 E4‐VEH versus E4‐GTA *p* = 0.7199) and the probe (6d) trial. The escape latency (*F* = 3.626, E4‐VEH versus E4‐GTA *p* = 0.0298), the percentage of time spent in the target quadrant (*F* = 7.202 E4‐VEH versus E4‐GTA *p* = 0.0295), the number of platform‐position crossings (*F* = 5.665, E3‐VEH versus E4‐VEH *p* = 0.9997, E3‐VEH versus E3‐GTA *p* = 0.0275 E4‐VEH versus E4‐GTA *p* = 0.0387), and the speed in the probe (6d) trial. *n* = 8, 9, 12, 12 mice for ApoE3‐VEH, ApoE3‐GTA, ApoE4‐VEH, and ApoE4‐GTA, respectively. Data are expressed as mean ± SEM. * *p* < 0.05 **p < 0.01, by two‐way ANOVA. Escape latency was evaluated by ANOVA for repeated measurement.

Human ApoE4 can cause age‐dependent impairments in learning and memory either in humans (F. Liu et al., 2010) or in knockin (KI) mice (Andrews‐Zwilling et al., 2010; Leung et al., 2012). Therefore, we speculated that Ac‐CoA supplementation may improve the synaptic function when alleviating the hippocampal aging in the elderly ApoE4‐TR mice. Transmission electron microscopy was performed to investigate the ultrastructure of the synapses in the hippocampal CA1 region, which detected a thinner postsynaptic density (PSD) in the 18‐month‐old ApoE4‐vehicle mice than in the age‐matched ApoE3‐vehicle mice. In addition, GTA treatment increased the number of synapses and the thickness and length of PSD in both ApoE3 mice and ApoE4 mice (Figure 4a). The synaptic plasticity‐related proteins were further quantified in the hippocampal synaptic extract (Figure 4b). Notably, the presynaptic proteins, including vesicular glutamate transporters 1 (VGLUT1) and synaptophysin (SYN), were significantly upregulated in the 18‐month‐old GTA‐treated ApoE4 mice. The postsynaptic proteins, including PSD95, NMDA receptor Type 2B (NR2B), and NMDA receptor Type 2A (NR2A), were obviously upregulated in the 18‐month‐old GTA‐treated ApoE3 and ApoE4 mice. Finally, Morris water maze (MWM) test showed that the spatial memory, including escape latency, number of crossings over the platform, and time in target quadrant, was significantly improved in the elderly GTA‐treated ApoE4 mice, which was also shown in the elderly GTA‐treated ApoE3 mice (Figure 4c). These results evidence that GTA administration ameliorates spatial cognition by improving the synaptic function in the elderly ApoE4 and ApoE3 mice.

## 4 DISCUSSION

Although cumulative evidence has demonstrated that *APOE* ε4 allele increases the risk for LOAD, the role of ApoE4 in brain aging remains unknown. The current study highlights that *APOE* ε4 allele may accelerate the senescence of hippocampal neurons by decreasing Ac‐CoA level and in turn lead to the cognitive impairment, which pinpoints an under‐recognized role of the *APOE* ε4 allele. In the study, we found that the hallmarks of aging, mTOR activation and endosome‐lysosome‐autophagy (ELA) system, were increased in the hippocampus of the elderly ApoE4 mice. Further metabolic and transcriptomic analyses, as well as biochemical analysis, reported an Ac‐CoA shortage in the hippocampus of the elderly ApoE4 mice. The Ac‐CoA supplementation ameliorated the hippocampal senescence of the elderly ApoE4 mice and significantly enhanced the synaptic structure and the spatial memory of these elderly ApoE4 mice.

The upregulation of senescence marker, activation of mTOR and ELA system are found in the hippocampus of aged ApoE4‐TR mice compared with control ApoE3‐TR mice, which only are reported previously in oncogene‐induced senescence (OIS) in vitro. Cellular senescence is a complex physiological and pathological phenomenon. Previous reports document that senescent cells are mainly located in neurons in the naturally aging brain (Chow et al., 2019) and in AD brain (Herdy et al., 2022). Consistently, in the current study, ApoE4 exacerbated cellular senescence in hippocampal neurons, as demonstrated in SA‐β‐Gal hyperstaining, upregulation of senescence marker gene and mTOR activation. Of note, the current study evidenced that enhanced ELA system exist in the hippocampus of the elderly ApoE4 mice. These results echo the findings that mTOR activation is accompanied by increased autophagy and SASP in OIS (Narita et al., 2011) and that the upregulated autophagy may promote the mTOR activation in senescent cells (Bernard et al., 2020).Currently, controversies remain regarding the role of autophagy in aging, with some studies claiming a reduced autophagy during aging (Jamshed et al., 2019) and some demonstrating an upregulated autophagic activity during aging (Tung et al., 2012). In the current study, although the autophagic upregulation was not pinpointed at the cellular level, our results clearly demonstrated that ApoE4 promoted the hippocampal senescence, as illustrated in mTOR activation and autophagy upregulation. Given these findings, we speculate that in certain situations, a reduction in autophagosomes may actually be beneficial to the aging cells.

Ac‐CoA is both a metabolite and a messenger molecule. In the current study, GTA supplementation (an FDA‐approved food additive) increased the total Ac‐CoA, thus obviously downregulating the senescent markers, mTOR activation, and ELA‐associated genes in the hippocampus of the elderly ApoE4 mice. These data suggest that Ac‐CoA deficiency is associated with autophagic upregulation, mTOR activation, and hippocampal neuronal senescence in the elderly ApoE4 mice, which is line with previous findings that Ac‐CoA inadequacy may enhance autophagy in lower organisms (Eisenberg et al., 2014; Zhu et al., 2020) and in cultured human cells and in mice (Zhu et al., 2020). Different from the previous study that increased the level of Ac‐CoA via the inhibition of Ac‐CoA carboxylase 1 (ACC1) with CMS121, thus providing neuroprotection and reducing the transcriptional markers of aging in SAMP8 (Currais et al., 2019), the current study directly increased the level of Ac‐CoA with its substrate, GTA, thus ameliorating the senescence in the hippocampus of the elderly ApoE4 mice. Of interest, after the GTA administration to ApoE3 mice, the levels of senescent markers and ELA‐associated genes did not change significantly, indicating that the energy state of cells or tissues themselves may affect their responsiveness to Ac‐CoA changes.

In addition, we found that GTA administration significantly improved spatial cognition through enhancing synaptic plasticity‐associated proteins in the elderly ApoE4 mice. Although the underlying mechanism was not further explored in the present study, we observed the age‐dependent decrease of Ac‐CoA in the hippocampus of the elderly ApoE4 mice and our previous study has demonstrated that GTA treatment can improve hippocampal plasticity in AD mice by increasing histone acetylation (H3K9 and H4K12) (Lin et al., 2023). We infer that the same mechanism may be involved in the Ac‐CoA‐improved senescence in the elderly ApoE4 mice. Transcriptomic and metabolic analysis revealed that the oxidative phosphorylation and lipid synthesis‐related pathways were significantly downregulated in the hippocampus of the elderly ApoE4 mice, which is consistent with the previous finding of a significant loss of oxidative phosphorylation and citric acid cycle (TCA) electron transport in senescent human neurons (Atamna, 2004). Of note, no difference was found in the activity of mitochondria complex I and complex IV. Instead, the activity of citric acid synthase drops in the first site of the TCA cycle. As age‐dependent decline in insulin signaling exists in the hippocampus of ApoE4 mice (N. Zhao et al., 2020), we analysis that the decrease in Ac‐CoA may result from a substrate deficiency during the process from glycolysis to the TCA cycle. Moreover, we used all female mice rather than male in present study, it is interested to dig out whether male mice could display such phenomena in future. Therefore, further studies are needed to verify these speculations.

## 5 CONCLUSION

Due to the deficiency of acetyl‐CoA during aging, ApoE4 exacerbates neuronal senescence, featuring an increased activation of mTOR and endosome‐lysosome‐autophagy system, which can be rescued by GTA supplementation. Interestingly, GTA treatment benefited both in ApoE3 and ApoE4 mice, particularly in ApoE4 mice, and consistently, expression of synaptic plasticity proteins was increased either in ApoE3 or in ApoE4 mice. These data suggest that GTA is beneficial in aged mice regardless of *APOE* genotype, which differs from previous reports that insulin (Reger et al., 2006; N. Zhao et al., 2017), pioglitazone (Abyadeh et al., 2021; Galimberti & Scarpini, 2017) and metformin (Zhang et al., 2019) affect cognitive function in aged mice in an *APOE* genotype‐dependent manner. These findings signify that GTA supplementation may serve as a promising therapeutic strategy for LOAD treatment.

## AUTHOR CONTRIBUTIONS

Xiaochun Chen and Jing Zhang conceived and designed the studies. Shuixin Lv and Yusi Zhang performed the experiments and analyzed data. Yingbin Lin detected mitochondrial complexes and synaptic protein. Wenting Fang quantified ATP level and NAD/NADH ratio. Yu Wang conducted SA‐β‐gal staining. Zihang Li and Anlan Lin performed the metabolic and transcriptomic analysis. Xiaoman Dai and Qinyong Ye talk about the data. Jing Zhang wrote and revised the manuscript. Xiaochun Chen interpreted the results and revised the manuscript. All author read and gave comments on the manuscript.

## FUNDING INFORMATION

This work was supported in part by the National Science Foundation of China (No. U21A20362, U22A20298, 82271468) and National Science and Technology Innovation 2030 Major Projects (No. 2022ZD0211600), and granted by the Joint Fund for Science and Technology Innovation of Fujian (No. 2018Y9095, No. 2018Y9057).

## CONFLICT OF INTEREST STATEMENT

The authors declare no competing interests.

## Supporting information


Figure S1

Figure S2

Table S1

## Data Availability

The raw sequencing files were available at Genome Sequence Archive (GSA, https://ngdc.cncb.ac.cn/gsa) following the GSA ID (CRA010762, CRA010748). The raw data of untargeted metabolism in OMIX, China National Center for Bioinformation (https://ngdc.cncb.ac.cn/omix accession no. OMIX004002). The data that support the findings of this study are available from the corresponding author upon reasonable request.
