# Supplementary figures and images for: ApoE4 exacerbates the senescence of hippocampal neurons and spatial cognitive impairment by downregulating acetyl‐CoA level
Source: Aging Cell. 2023 Aug 18;22(9):e13932. doi: 10.1111/acel.13932 (PMC10497817; doi:10.1111/acel.13932)

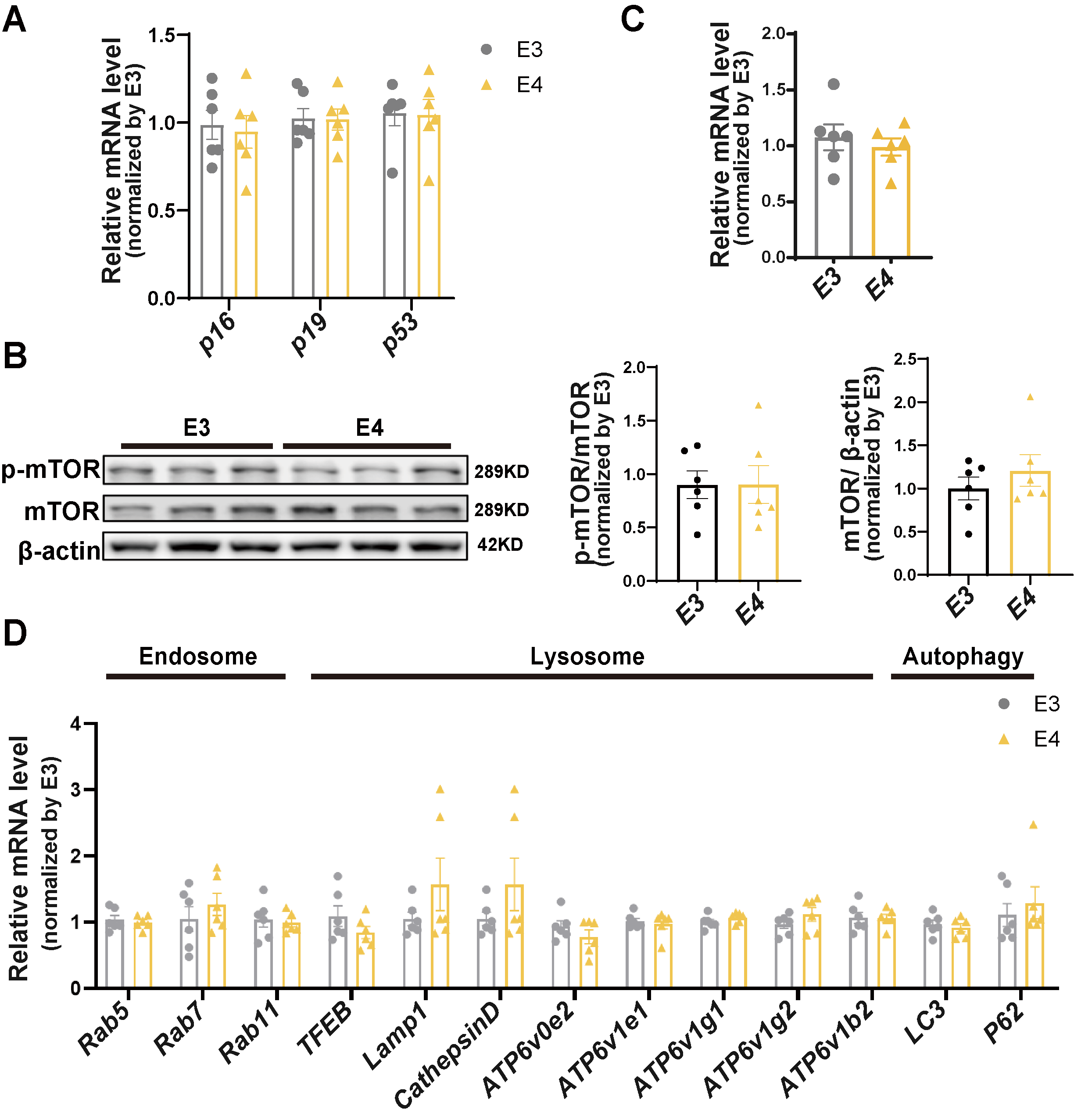

Supplement: Supplementary file 1 — Figure S1 [file ACEL-22-e13932-s003.png]

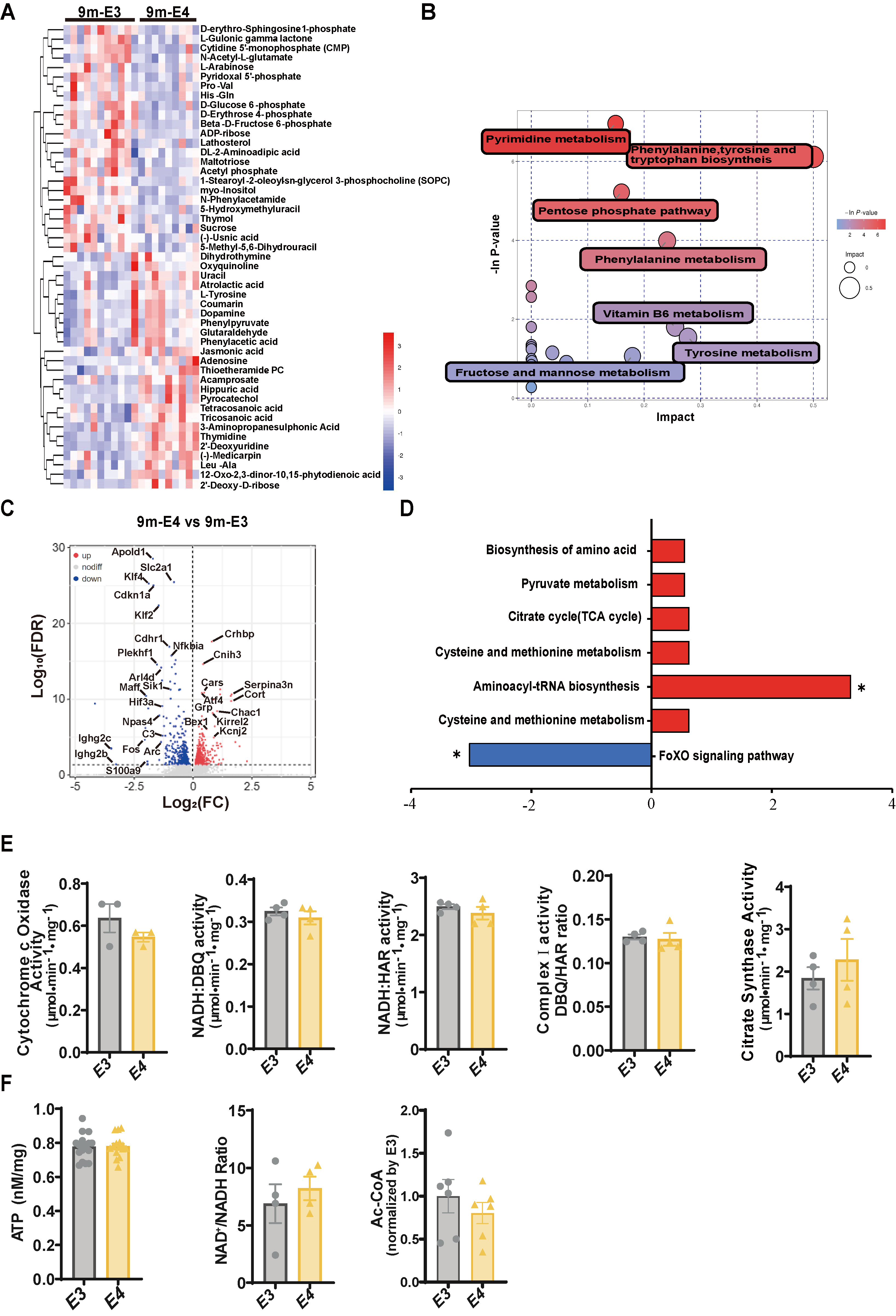

Supplement: Supplementary file 2 — Figure S2 [file ACEL-22-e13932-s001.png]
