# Supplementary material for: ApoE4 exacerbates the senescence of hippocampal neurons and spatial cognitive impairment by downregulating acetyl‐CoA level
Source: Aging Cell. 2023 Aug 18;22(9):e13932. doi: 10.1111/acel.13932 (PMC10497817; doi:10.1111/acel.13932)
Supplement: Supplementary file 3 — Table S1 [file ACEL-22-e13932-s002.docx]

**Supplemental Table 1**

| Gene name | Forward primer（5’→3’） | Reverse Primer（5’→3’） |
| --- | --- | --- |
| p16 | ACATCAAGACATCGTGCGATATT | CCAGCGGTACACAAAGACCA |
| p19 | CTGAACCGCTTTGGCAAGAC | GCCCTCTCTTATCGCCAGAT |
| p53 | GATGACTGCCATGGAGGAGT | GTCCATGCAGTGAGGTGATG |
| mTOR | ACCGGCACACATTTGAAGAAG | CTCGTTGAGGATCAGCAAGG |
| Rab5 | GGACGTGGGGAATCCTCTGA | AGAAGCCGGAGAAATCAAACC |
| Rab7 | AAGCCACAATAGGAGCGGAC | AGACTGGAACCGTTCTTGACC |
| Rab11 | CTCTGGACGAGGTCTTCCG | TGTTCCGTGTGAACTGGATGG |
| TFEB | CCACCCCAGCCATCAACAC | CAGACAGATACTCCCGAACCTT |
| LAMP1 | CAGCACTCTTTGAGGTGAAAAAC | ACGATCTGAGAACCATTCGCA |
| Cathepsin D | CCTGGCTTCGTCCTCCTTC | GGCGATGACTGCATGGAGT |
| ATP6v0e2 | CTGGTTCGTGCCCAAAGGA | GGCAATGAGCCAGAAGAGGTAA |
| ATP6v1e1 | GAATCAAGCAAGGCTCAAAGTCC | CGGGTCGTATCTTTTACCACC |
| ATP6v1g1 | CCCAGGCTGAAATTGAACAGT | TTCTGGAGGACGGTCATCTTC |
| ATP6v1g2 | GAGGAGGCTCAAATGGAGGTG | CTGAACCTGCCGTCTTGTG |
| ATP6v1b2 | TTTCCCAGATACGCTGAGATTGT | GGAGCCACTAACTTCTAGGACT |
| LC3 | GACCGCTGTAAGGAGGTGC | CTTGACCAACTCGCTCATGTTA |
| P62 | ATGTGGAACATGGAGGGAAGA | GGAGTTCACCTGTAGATGGGT |
| IL6 | TAGTCCTTCCTACCCCAATTTCC | TTGGTCCTTAGCCACTCCTTC |
| Casapase 8 | TGCTTGGACTACATCCCACAC | TGCAGTCTAGGAAGTTGACCA |
| Pai | GTGAATGCCCTCTACTTCAGTG | GCTGCCATCAGACTTGTGGAA |
| β-actin | GGTCATCACTATTGGCAACG | TCCATACCCAAGAAGGAAGG |
